# Editorial: Novel biomarkers for anticancer therapy

**DOI:** 10.3389/fphar.2022.1109761

**Published:** 2023-01-06

**Authors:** Ting Wang, Zhe-Sheng Chen, Chen Shi, Liang Ouyang, Hongtao Xiao

**Affiliations:** ^1^ Department of Clinical Research, Sichuan Cancer Hospital and Institution, Sichuan Cancer Center, School of Medicine, University of Electronic Science and Technology of China, Chengdu, China; ^2^ Department of Pharmaceutical Sciences, College of Pharmacy and Health Sciences, St. John’s University, Queens, NYC, United States; ^3^ Department of Pharmacy, Union Hospital, Tongji Medical College, Huazhong University of Science and Technology (HUST), Wuhan, China; ^4^ Hubei Province Clinical Research Center for Precision Medicine for Critical Illness, Wuhan, China; ^5^ State Key Laboratory of Biotherapy, Sichuan University-University of Oxford Huaxi Joint Centre for Gastrointestinal Cancer, West China Hospital, Sichuan University, Chengdu, China; ^6^ Department of Pharmacy, Sichuan Cancer Hospital and Institution, Sichuan Cancer Center, School of Medicine, University of Electronic Science and Technology of China, Chengdu, China

**Keywords:** biomarker, anticancer, non-coding RNAs, chemoresisitance, gene mutation, metabolic reprogramming

Cancer is a serious chronic disease that poses a great threat to human health worldwide. Despite the rapid development of targeted therapy and immunotherapy in recent years, the diagnosis and treatment of cancer are still facing huge challenges due to high tumor heterogeneity, a lack of typical biomarkers, and therapeutic resistance. Therefore, it is of great significance to reveal novel and reliable biomarkers in cancer; illustrate the mechanisms of cancer occurrence, development, and therapeutic resistance; and develop new anticancer drugs with high efficacy and low toxicity. The Research Topic titled “*Novel Biomarkers for Anticancer Therapy*” was opened between 21 January 2022 and 21 July 2022. Finally, a total of 32 articles (8 reviews, 5 systemic reviews, and 19 original articles) were accepted in 90 submissions, providing a new and promising direction for future anticancer therapy.

Non-coding RNAs (ncRNAs) are emerging RNA molecules that have been found to play essential roles in cancer progression and therapeutic resistance. In recent decades, ncRNAs have gradually attracted the attention of researchers worldwide. In this Research Topic, five articles focus on lncRNAs, an ncRNA that has more than 200 nucleotides and various functions (Tan et al., 2021). Feng et al. found that the lncRNA ENST869 was upregulated in MCF-7 and MDA-MB-231 cells treated with chidamide. Subsequent experiments suggested that lncRNA ENST869 could promote nestin transcription by binding to the nestin transcriptional region, thus affecting the sensitivity of breast cancer to chidamide. Xiao et al. explored the role of lncRNA HAGLROS in bladder cancer (BC) and discovered that HAGLROS could facilitate BC progression by modulating the miR-330-5p/SPRR1B axis. Additionally, the upregulation of lncRNA PRADX induced by the UNX1-CBFβ complex can accelerate energy metabolism, leading to expediting mesenchymal glioblastoma (GBM) development and poor prognosis in mesenchymal GBM patients (Xu et al.). A pan-cancer analysis revealed that LINC00857 was upregulated in various tumors and associated with an immunosuppressive microenvironment, implying that LINC00857 might serve as a valuable biomarker and therapeutic target for immunotherapy (Ren et al.). Zhang et al. identified genomic instability-related lncRNAs (GIRlncRNAs) in non-small cell lung cancer (NSCLC) and subsequently constructed a risk-scoring model based on a GIRlncRNA signature, contributing to the future of personalized treatment for NSCLC. In addition to lncRNAs, another essential class of ncRNAs, microRNAs (miRNAs), also have momentous effects on the proliferation, migration, and invasion of tumor cells. Yan et al. summarize the latest advances of miRNAs in therapeutic resistance in NSCLC and discuss their potential clinical values.

Chemotherapy, as an important cancer treatment, can effectively reduce the recurrence and metastasis of many malignant tumors. However, many negative factors, such as larger adverse reactions and drug resistance, seriously hinder the clinical use of chemotherapeutic drugs. Zhou et al. compare the adverse reactions of olaparib in the treatment of three types of cancer *via* meta-analysis, showing that the adverse reactions of olaparib in the treatment of different cancers are themselves different and suggesting that clinicians should conduct a specific analysis of the treatments of different cancers. Recent evidence uncovered by Du et al. suggests that cathepsin L could reduce the sensitivity of neuroblastoma to cisplatin and doxorubicin by elevating the expression of SRGN. In addition, Xu et al. found that the overexpression of RAB39B could augment doxorubicin and vincristine resistance in diffuse large B-cell lymphoma (DLBCL). Yang et al. conducted a multicenter study to predict the clinical characteristics, survival outcomes, and prognosis of elderly patients with DLBCL. This study could help doctors to provide more appropriate treatment options for elderly patients with DLBCL. Nine hub genes, which may modulate NSCLC progression and cisplatin resistance, were identified by Mengyan et al., but more experiments are needed to verify their conclusion.

In recent years, tremendous advances in targeted therapy and immunotherapy have brought hope of survival to many cancer patients. Vemurafenib, a targeted anticancer drug, has been approved for the treatment of BRAF-mutated melanoma patients (Kramkimel et al., 2016). Wu et al. found that RSK2 could interact with FOXO1 to elevate cyclin D1 expression, resulting in the upregulation of cell proliferation and vemurafenib resistance in melanoma. It was found that fibroblast growth factor receptors (FGFRs) are potential targets for gastric cancer. Notably, Zeng et al. synthesized a novel FGFR inhibitor RK-019 and demonstrate its anticancer activity *in vitro* and *in vivo*.

Furthermore, Cheng et al. performed a comprehensive analysis of 15 efferocytosis-associated molecules and 12 immune checkpoint-related molecules in 16 cancer types, including the construction of a protein-protein interaction network, differential expression analysis, correlation analyses with overall survival (OS), tumor microenvironment, and anticancer drug sensitivity. Finally, they conclude that targeting these molecules might be instrumental to overcoming drug resistance and improving patient outcomes. Immunotherapy based on immune checkpoint inhibitors (ICIs) has seen rapid development in the field of clinical cancer treatment. Li et al. reviewed the action mechanisms and clinical trials of PD-1/PD-L1 inhibitors and approved PD-1/PD-L1 inhibitors in advanced NSCLC. Additionally, four meta-analyses about the clinical use of ICIs were included in our Research Topic. In one of these studies, the clinical outcomes of urothelial cancer patients who were simultaneously treated with ICIs and proton pump inhibitors (PPIs) were predicted, showing that concomitant PPI use led to increased progression and death risk and a decreased objective response rate (Zhang et al.). Moreover, Ye et al. clarify that sintilimab could significantly improve OS and progression-free survival (PFS) compared with other anticancer drugs. Sintilimab might become a promising treatment strategy for cancer. Many studies have shown that chemotherapy was not only cytotoxic but also had an effect on activating the anti-tumor immune response, implying that chemotherapy, combined with immunotherapy, may be an effective strategy for the treatment of malignant tumors (Wu and Waxman, 2018). Chen et al. unveil that chemotherapy combined with immunotherapy brings better survival benefits but a higher risk of adverse reactions for squamous NSCLC patients compared with the chemotherapy group.

An altered tumor microenvironment (TME) is highly likely to affect tumor progression, such as tumor metastasis and immunotherapy efficacy. Solute carrier family 11, member 1 (SLC11A1), related to TME, was reported to be positively correlated with immune cell infiltration, including macrophage and fibroblast, and its high expression could reduce immunotherapy benefits in colorectal cancer (CRC). At the end of the article, the authors conclude that SLC11A1 may serve as a promising biomarker and therapeutic target in CRC (Ma et al.). In another study analyzed from a pan-cancer perspective, Ma et al. found that CTT3 depletion could promote immune cell infiltration and some immune checkpoint gene expressions, including CD274, PDCD1, and CTLA4. Additionally, TP53, KRAS, and some epigenetic factors were found to play essential roles in the high expression of CTT3. Interestingly, accumulating studies have shown that a novel form of cell death called ferroptosis could influence immunotherapy efficacy by regulating TME. Therefore, the identification of ferroptosis-related prognostic genes in ovarian cancer (OV) could help to improve prognoses and promote precision immunotherapy, posing benefits to OV patients (Liu et al.).

Gene mutation exerts a critical role in carcinogenesis and cancer development. A comprehensive and deeper understanding of gene mutation exerted in cancer aids cancer diagnosis and treatment. A study performed by Liu et al. explores the correlation between KRAS/EGFR/TP53 single gene mutation and the survival benefits of cancer patients treated with ICIs. Their results show that the OS and PFS of patients with KRAS, EGFR, or TP53 mutation were substantially elevated, suggesting that the detection of genetic mutations in patients will help to predict the clinical benefits of patients receiving ICIs. Hepatoid adenocarcinoma of the lung (HAL) is an infrequent tumor and lacks unified diagnostic standards, resulting in late diagnosis and poor prognosis. Yao et al. discovered two driver mutation genes and nine potential driver mutation genes in HAL, providing a guide for the diagnosis and treatment of this rare malignant tumor for future use. CRISPR/Cas9, as a novel and efficient gene editing technology, has motivated the rapid development of research about cancer initiation, progression, and drug resistance. Ongoing clinical trials based on CRISPR/Cas9 are providing therapeutic strategies for cancer related to gene mutation (Chen et al.).

Metabolic reprogramming is one of the vital hallmarks of cancers. Tumor cells are able to meet their survival needs in some adverse environments by altering their metabolism. Research focused on energy metabolism is currently ongoing. Yan et al. performed a metabolomics analysis in NSCLC patients receiving epidermal growth factor receptor tyrosine kinase inhibitors (EGFR-TKIs) or PD-1/PD-L1 inhibitors and found that EGFR-TKIs and PD-1/PD-L1 inhibitors could promote lipid and amino acid metabolism alterations, providing a new direction for elucidating the antitumor mechanisms of targeted therapy and immunotherapy. Lipids not only act as biological membrane components but also as signaling molecules in many key signaling pathways and essential sources of cellular energy (Bian et al., 2021). A lot of regulatory molecules, which participate in lipid metabolism, exhibit significant roles in the proliferation, migration, and invasion of tumor cells. Four reviews respectively summarize the roles of fatty acid-binding proteins (Sun et al.), acyl-CoA synthetase long-chain family 4 (Hou et al.), Niemann-Pick C1-like 1 (Zhang et al.), and hydroxy Acyl-CoA Dehydrogenase (Fang et al.) in cancer, including their structures, distribution, functions, and clinical significance. Thus, these molecules might be ideal therapeutic targets in cancer treatment.

Early screening, diagnosis, and treatment of tumors can greatly improve prognoses and reduce mortality. Therefore, it is vital to identify typical and reliable biomarkers in malignant tumors. Niu et al. discovered two markers (haptoglobin and protein disulfide-isomerase A3) in the serum of patients with CRC. They may become ideal tumor markers like traditional carcinoembryonic antigens and carbohydrate antigens (19-9) in CRC. Furthermore, Wang et al. reveal the roles of Fraser syndrome protein 1(FRAS1)/fras1-related extracellular matrix protein (FREM) family members in kidney clear cell renal cell carcinoma. As described by them, FRAS1/FREM family members could act as dependable predictors of cancer progression and targeted therapeutic drug response in kidney clear cell renal cell carcinoma. Xu et al. sum up the effects of chemokines and their receptors in cancer and look at them as significant markers in the future of screening, diagnosis, and treatment of tumors.

In summary, the “*Novel Biomarkers for Anticancer Therapy*” Research Topic collects studies focused on finding novel biomarkers and therapeutic targets in cancer, paving the way for developing novel therapeutic strategies and overcoming therapeutic resistance. We hope that accumulating research associated with carcinogenesis, cancer progression, and therapeutic resistance will help improve survival outcomes and prognoses for cancer patients in the future.
